# The War and Our Supply of Drugs

**Published:** 1917-03-24

**Authors:** 


### THE WAR AND OUR SUPPLY OF DRUGS.

At a recent meeting of the Royal Society of Arts Mr. F. A. Hocking, B.Sc., pharmaceutist to the London Hospital, read a paper on "The War and Our Supply of Drugs." The subject was divided into three sections, in which were considered: (1) What drugs were in use before the war, and to what extent we were dependent upon Central Europe for their supply. (2) The difficulties arising out of the war, and to what extent these have been surmounted. (3) The probable position after the war.

Mr. Hocking submitted that the practice of the great general hospitals of to-day is a guide in determining what drugs helpful and necessary to the treatment of disease will be used in the private practice of to-morrow. He pointed out that times are changing; the physician formerly derived his principal remedies from the vegetable kingdom, now new methods of treatment have been devised which do not necessarily involve the use of drugs at all, such as the employment of vaccines, of physical agents as light, electricity, etc., and treatment by means of gymnastics, radio-activity, and so forth. The greatly extended use of substances of definite chemical composition derived from the mineral kingdom or prepared in the chemical laboratory has tended to reduce the use of vegetable drugs. For these and other reasons it comes about that many of the drugs described in a text-book of vegetable materia medica are now rarely prescribed.

The list of drugs actually used in the London Hospital in 1914 included eighty of vegetable origin. Of these very few indeed were derived from Central Europe. The chief medicinal plants formerly procured from enemy countries were aconite-root, belladonna root and leaves, colchicum-corm, digitalis-leaves, gentian-root, henbane-leaves, opium, and possibly valerian-root. After alternative sources of supply had been sought and substitutes in some cases found acceptable, difficulties remained only in the cases of belladonna root and leaves, hyoscyamus-leaves, and valerian. In respect of belladonna-root the London Hospital obtained 1 cwt. of excellent (wild) root from Hampshire. To solve remaining difficulties the business enterprise of the proprietors of the old-established drug-farms of this country to meet in some degree the problem arising out of the present situation will no doubt be helpful.

Mr. Hocking dealt with the origin of animal substances, such as lanolin, of alkaloids, acids, compounds of the commoi metals, "organic chemicals," and "synthetics," and considered their importance and the possibility of obtaining indopendence in supply. Having sketched the position before the war, the paper next dealt with the difficulties arising out of the war, the outbreak of which was accompanied by an outbreak of profiteering. Many dealers threw patriotism to the winds, and seized every opportunity of gaining control of existing stocks and of the future output of home manufacturers, repudiating contracts, and then proceeding to make money out of their customers' embarrassments. Manufacturers on the other hand, loyally fulfilled their contracts, even in some cases at a loss to themselves. Real difficulties were comparatively few, but very formidable. These were associated mainly with the supply of raw material, particularly of potash and of bromine, and the supply of synthetic and some other drugs. In respect of bromine the blockade left the Americans masters of the situation—a fact of which they were not slow to take advantage, for at one time the price of potassium bromide was 25s. per lb., as compared with 1s. 6½d. per lb. in July 1914.

In respect of the period after the war it was suggested that the scientists of these islands are fully equal to the solution of all questions relating to synthetic drugs, and the hands of manufacturers will not be tied in respect of provision of plant and labour. To compete with cheap foreign production, and to continue manufactures established in this country during the last two years, some form of State aid probably will be necessary.

